# The Groove Enhancement Machine (GEM): A Multi-Person Adaptive Metronome to Manipulate Sensorimotor Synchronization and Subjective Enjoyment

**DOI:** 10.3389/fnhum.2022.916551

**Published:** 2022-06-15

**Authors:** Lauren K. Fink, Prescott C. Alexander, Petr Janata

**Affiliations:** ^1^Center for Mind and Brain, University of California, Davis, Davis, CA, United States; ^2^Neuroscience Graduate Group, University of California, Davis, Davis, CA, United States; ^3^Department of Music, Max Planck Institute for Empirical Aesthetics, Frankfurt am Main, Germany; ^4^Max Planck – NYU Center for Language, Music, and Emotion (CLaME), Frankfurt am Main, Germany; ^5^Center for Neuroscience, University of California, Davis, Davis, CA, United States; ^6^Department of Psychology, University of California, Davis, Davis, CA, United States

**Keywords:** auditory feedback, tapping, social, individual differences, open-source, assistive device

## Abstract

Synchronization of movement enhances cooperation and trust between people. However, the degree to which individuals can synchronize with each other depends on their ability to perceive the timing of others’ actions and produce movements accordingly. Here, we introduce an assistive device—a multi-person adaptive metronome—to facilitate synchronization abilities. The adaptive metronome is implemented on Arduino Uno circuit boards, allowing for negligible temporal latency between tapper input and adaptive sonic output. Across five experiments—two single-tapper, and three group (four tapper) experiments, we analyzed the effects of metronome adaptivity (percent correction based on the immediately preceding tap-metronome asynchrony) and auditory feedback on tapping performance and subjective ratings. In all experiments, tapper synchronization with the metronome was significantly enhanced with 25–50% adaptivity, compared to no adaptation. In group experiments with auditory feedback, synchrony remained enhanced even at 70–100% adaptivity; without feedback, synchrony at these high adaptivity levels returned to near baseline. Subjective ratings of being in the groove, in synchrony with the metronome, in synchrony with others, liking the task, and difficulty all reduced to one latent factor, which we termed enjoyment. This same factor structure replicated across all experiments. In predicting enjoyment, we found an interaction between auditory feedback and metronome adaptivity, with increased enjoyment at optimal levels of adaptivity only with auditory feedback and a severe decrease in enjoyment at higher levels of adaptivity, especially without feedback. Exploratory analyses relating person-level variables to tapping performance showed that musical sophistication and trait sadness contributed to the degree to which an individual differed in tapping stability from the group. Nonetheless, individuals and groups benefitted from adaptivity, regardless of their musical sophistication. Further, individuals who tapped less variably than the group (which only occurred ∼25% of the time) were more likely to feel “in the groove.” Overall, this work replicates previous single person adaptive metronome studies and extends them to group contexts, thereby contributing to our understanding of the temporal, auditory, psychological, and personal factors underlying interpersonal synchrony and subjective enjoyment during sensorimotor interaction. Further, it provides an open-source tool for studying such factors in a controlled way.

## Highlights

– To aid people in synchronizing with each other, we built an assistive device that adapts in real-time to groups of people tapping together.
– By varying the adaptivity of a metronome, we show that we can enhance group synchrony and subjective feelings of enjoyment.
– Both individuals and groups benefit from an optimally adaptive metronome, regardless of their previous musical experience.
– Auditory feedback about one’s own and others’ taps influences both motor synchrony and subjective experience, and interacts with metronome adaptivity.
– The multi-person adaptive metronome allows to study, in a controlled way, the factors that influence interpersonal synchronization and social bonding.

## Introduction

Sensorimotor synchronization (SMS)—the temporal alignment of motor behavior with a rhythmic sensory stimulus—has been observed in a variety of species and sensory modalities (Greenfield, 2005; Ravignani et al., 2014). Among humans, SMS has been shown to enhance prosocial behavior, social bonding, social cognition, perception, and mood—both within groups and toward outsiders [see Mogan et al. (2017) for review]. While sensorimotor synchronization can occur in a variety of contexts, we focus here specifically on auditory-motor coupling, which has been studied extensively in the music cognition literature as a critical mechanism of musical engagement and prosocial behavior.

When humans interact together in motoric synchrony, they are more likely to subsequently exhibit cooperative behavior, successful joint actions, trust of others, and altruism (Wiltermuth and Heath, 2009; Valdesolo et al., 2010). While such benefits occur during pure motor synchrony—for example, walking in step together—using music to organize movement is a powerful temporal cue and culturally relevant activity. The use of music, or even just a metronome, can enhance cooperative behavior—typically measured via economics games, such as the Public Goods Game (Wiltermuth and Heath, 2009; Kniffen et al., 2017) or Prisoner’s Dilemma (Anshel and Kipper, 1988)—and feelings of synchronization or connection with others, typically measured via self-report surveys (Hove and Risen, 2009; Fairhurst et al., 2013, 2014; Zhang et al., 2016; Kirschner and Tomasello, 2010). Interestingly, these prosocial effects are not specific to adults (Kirschner and Tomasello, 2010). Infants as young as 14-months show increased helping behavior toward adults who have bounced together with them synchronously to music (Cirelli et al., 2014a). Such helping behavior even generalizes to positive affiliates of the adults, i.e., adults the infants had seen interact together, though not toward neutral strangers (Cirelli et al., 2014b).

Importantly, however, for music to benefit motoric synchrony and interpersonal coordination, those engaging with it must be able to extract its temporal regularity. Generally, the beat and its (sub)harmonics are the relevant periodicities to which those engaging with music synchronize their movements. Because the perceived beat is typically isochronous (Merker et al., 2009) or quasi-isochronous (Merchant et al., 2015), many studies of sensorimotor synchronization involve asking individual participants to tap to an isochronous auditory pulse or beat (i.e., tap with a metronome), as such a task contains the most basic elements of what occurs during engagement with more complex music. However, the degree to which individuals can perceive and align their action to an auditory pulse varies among individuals and may impede their ability to synchronize well with others.

The most basic version of a sensorimotor synchronization task involves having a single participant synchronize their finger taps with a metronome. These single-person isochronous tapping experiments have revealed much about the motor and cognitive processes underlying sensorimotor synchronization. In terms of motor constraints, the lower inter-onset-interval (IOI) limit for a single finger tapping in 1:1 synchrony with an auditory pulse is around 150–200 ms, though highly trained musicians and/or bimanual tapping may result in slightly lower IOIs of around 100 ms [see Repp (2005) for review]. On the other end of the spectrum, a perceptual constraint prohibits participants from being able to synchronize their taps with IOIs longer than ∼1.8 s. Such durations become too hard to predict accurately; participants’ taps become reactive to the metronome tones, rather than anticipatory (Repp, 2005; c.f., Repp and Doggett, 2007). Though these studies of IOI constraints have typically been conducted with adult participants, a growing number of developmental studies (van Noorden and De Bruyn, 2009; Provasi et al., 2014; Thompson et al., 2015) have found that synchronizing with slower tempi becomes less difficult with age. With these limitations in mind, in the following experiments, we have adults stay within a comfortable range of synchronization tempi, using a starting tempo of 120 beats per minute (an IOI of 500 ms).

Complementing tapping tasks using invariant (strict) metronomes set at different tempi, studies seeking to understand the dynamics underlying more realistic joint musical interactions (Repp and Keller, 2008; Fairhurst et al., 2013) have used metronomes that adapt their timing based on the asynchronies between the metronome’s tone and the participant’s tap. The idea behind an adaptive metronome is that it mimics, in a controlled way, the adaptive behavior another human might adopt during joint tapping. For example, Fairhurst et al. (2013) used a personal computer-based adaptive metronome, implemented in MAX / MSP, that adapted each subsequent metronome tone by some percentage (0, 25, 50, 75, or 100%) of the participant’s tap asynchrony relative to the current tone, while they were in a magnetic resonance imagining (MRI) scanner. They found that, across sets of taps (20 taps/trial), adaptive metronome settings of 25% and 50% brought participants into greater synchrony with the metronome, compared to a non-adapting metronome. The opposite was also true; a metronome adapting by 75% or 100% significantly worsened synchronization performance. Functional MRI analyses revealed distinct brain networks differentially activated when participants are in vs. out of synchrony. Greater synchrony resulted in increased motor and “Default Mode Network” activity, possibly related to the social and effortless aspects associated with being “in the groove (Janata et al., 2012),” whereas poor synchrony resulted in increased activity in cognitive control areas of the brain, likely reflecting the increased effort required to align taps with the metronome. Given this ability to enhance or perturb sensorimotor synchronization and subjective experience using an adaptive metronome—coupled with the known group benefits of synchronous motor action outlined previously—we sought, in the current study, to extend the use of an adaptive metronome to a multi-person context.

To our knowledge, the current study is the first to utilize an adaptive metronome in a group-tapping context; however, recent work has explored synchronization among groups of individuals [see Repp and Su (2013), Part 3, for review]. Such studies range from examinations of synchronization in musical ensembles (Rasch, 1979), or duetting pianists (Goebl and Palmer, 2009; Zamm et al., 2015; Demos et al., 2019), to those of how synchrony affects social affiliation between two tappers (adults: Hove and Risen, 2009; Kokal et al., 2011; children+adult: Rabinowitch and Cross, 2018). Additionally, others have explored important factors, such as auditory and visual feedback, that influence tapping synchrony. With respect to auditory feedback, Konvalinka et al. (2010) showed coupling between dyadic tappers changes as a function of auditory feedback and participants are actually worst at keeping the tempo when they can hear each other. More recent evidence suggests such a result might be mediated by musicianship (Schultz and Palmer, 2019); musicians perform well with self and other feedback but non-musicians are worse when receiving feedback from others’ taps. In a study of triads with one leader, two followers, and varied conditions of cross-participant feedback, Ogata et al. (2019) show the effect of auditory feedback is dependent on the assigned leader-follower roles. Using visual feedback, Tognoli et al. (2007) observed that seeing the other tapper’s finger induced spontaneous synchronization during self-paced finger tapping (without a metronome). Additionally, Timmers et al. (2020) recently showed that visual information reduced synchronization accuracy during dyadic co-performance (one participant live, one participant recorded).

Considering these studies, especially in conjunction with the results of single-person tapping tasks, it is clear that audio and/or visual feedback has an effect on tapping performance. Nonetheless, it remains unclear whether such effects manifest among multiple individuals when the metronome is adaptive. Thus, in the following experiments, we manipulated all versions of auditory feedback (hearing only the metronome, hearing only the metronome and oneself but not others in the group, and hearing the metronome, oneself, and everyone else in the group). So as to avoid visual cues influencing synchronization, we asked participants to look only at their own tapping finger.

The main questions posed in our experiments were: (1) can group synchrony (both objective and subjective) be enhanced using an adaptive metronome? And (2) does auditory feedback influence group synchrony and subjective experience? To address these questions, we introduce a novel hardware/software system for perturbing a metronome and collecting multi-person tapping data in a highly customizable, temporally precise way. This research thus extends the previous adaptive metronome paradigm of Repp and Keller (2008) and Fairhurst et al. (2013) to group contexts.

### The Current Study

We created an adaptive device to assist individuals or groups tasked with synchronizing to a pulse. While a variety of hardware and software has been developed for tapping experiments, none fill the exact niche of our device. For example, the E-music Box (Novembre et al., 2015) is a group music-making system giving participants the ability to control the output timing of a musical sequence based on their cyclical rotary control of an electromagnetic music box. Our device, on the other hand, adapts to the measured asynchrony of an individual participant’s tap, or to the mean asynchrony of a group of participants tapping together, relative to a defined metronome period. While other systems have been developed to collect and analyze tapping data, such as FTAP (Finney, 2001), MatTAP (Elliott et al., 2009), Tap-It (Kim et al., 2012), Tap Arduino (Schultz and van Vugt, 2016), and TeensyTap (van Vugt, 2020), none of these are currently capable of adaptive, group-tapping experiments.

In building the multi-person adaptive metronome, we sought to keep latencies in the system to a minimum. We were inspired by Tap Arduino (Schultz and van Vugt, 2016), which uses a force-sensitive resistor (FSR) as a tap pad connected to an Arduino microcontroller and PC to collect tapping data. In the validation of Tap Arduino, Schultz and van Vugt (2016) showed that the average latency of the Arduino-based tap pad is less than 3 ms, significantly lower than latencies produced by a standard percussion pad (∼9 ms), FTAP (∼15 ms), and MAX/MSP (∼16 ms) (Cycling’74, 2014). Hence, we decided to build our device using multiple Arduino Uno microcontrollers and FSRs. Please see additional details below in *Apparatus.*

In order to better understand the sensorimotor synchronization of groups in the presence of an adaptive metronome, we conducted five tapping experiments. The goal of the first experiment was to replicate Fairhurst et al. (2013). Therefore, the experiment involved single tappers. The metronome played aloud through a speaker and adapted to participant performance using positive phase correction, as in experiment 1 of Repp and Keller (2008) and Fairhurst et al. (2013), with the following equation:


(1) $$t_{met_{n+1}}=t_{{\text{met}}_{n}}+IOI_{met}+\left(\alpha \times async_{n}\right)$$

where the time of the upcoming metronome tone (t_met n+1_) is equal to the time of the current metronome tone, plus the metronome inter-onset-interval (IOI), plus an adaptation based on the human participant’s asynchrony. The asynchrony (async_n_) is defined as the participant’s tap time minus the metronome tone time (t_tap_n__ – t_met_n__). Alpha (α) represents the adaptivity parameter, and is a fractional multiplier of the asynchrony. Note, if the participant fails to tap, async_n_ = 0; the next metronome tone will, therefore, occur at the default IOI. Alpha can be set to any number. When set to α = 0, the metronome does not adapt, and is thus considered a control (reference) condition. In Fairhurst et al. (2013), alpha values of 0.25 and 0.5 were found to be optimally adaptive, resulting in a smaller average standard deviation of asynchronies than with a non-adaptive metronome, while values of 0.75 and 1 were overly adaptive, leading to larger SD asynchronies.

Because we were also interested in the effects of auditory feedback on tapping stability, in Experiment 2 we performed an additional version of the single-person experiment outlined above in which participants now heard the sound triggered by their own tap through headphones (the metronome still played aloud through a speaker). We expected the findings from tapping studies with auditory feedback and a standard metronome (e.g., Aschersleben and Prinz, 1995; Aschersleben, 2002) to replicate, such that participants would become more accurate when they received auditory feedback from their taps. We further hypothesized that this greater objective synchrony (tapping accuracy) would correspond to increased ratings of feeling in the groove, liking of the task, etc.

Experiments 3–5 involved groups of four tappers. In these cases, the metronome algorithm was adjusted to adapt based on the average asynchrony of the group (of size I; in our case, 4 tappers):


(2) $$t_{met_{n+1}}=t_{met_{n}}+IOI_{met}+\left(\alpha \times \frac{1}{I}\sum_{i=1}^{I}async_{\text{n\_i}}\right)$$

with all terms as previously defined. In Experiment 3, participants heard the sound of the metronome through its speaker but not the sound of their own tap. In Experiment 4, participants heard the metronome through its speaker and only the sound of their own tap through headphones. In Experiment 5, participants heard the metronome through its speaker and the sounds of their own and everyone else’s taps through speakers attached to each individual Arduino.

Across all experiments, we hypothesized that, compared to no adaptivity or highly adaptive conditions, low levels of metronome adaptivity (i.e., 25–50%) would result in more stable tapping performance, as well as a more positively valanced subjective experience. We further hypothesized that participants’ being able to hear their taps more clearly in Experiments 2, 4, and 5 would result in greater temporal accuracy than in Experiments 1 and 3 (in which they could not hear a sound produced by their taps). With regard to Experiment 5, we hypothesized participants would feel more connected to and in synchrony with the group when they could hear each other’s taps. A summary of experimental conditions and hypotheses is provided in Table 1.

**TABLE 1:** Summary of experiments, conditions, and hypotheses.

Exp.	# tappers	Tap audio feedback	α levels	Hypothesis
1	1	None	0, 0.25, 0.5, 0.75, 1	Replicate Fairhurst et al. (2013)
2	1	Headphones	0, 0.25, 0.5, 0.75, 1	Reduced async due to audio feedback
3	4	None	0, 0.35, 0.7, 1	Benefits of adaptive metronome extend to group
4	4	Headphones	0, 0.35, 0.7, 1	Reduced async due to audio feedback
5	4	Speakers	0, 0.35, 0.7, 1	Reduced async; increased perceived group sync

*Alpha (α) levels correspond to metronome adaptivity conditions; 0 means no adaptivity (control condition, i.e., standard metronome).*

*Experiment 5 is the only experiment in which participants could hear the taps produced by other tappers.*

*async, asynchrony; # tappers, the number of participants together in the experiment session; Tap audio feedback, whether or not participants could hear the sound triggered by their tapping on the FSR and how.*

#### Apparatus

**FIGURE 1:**
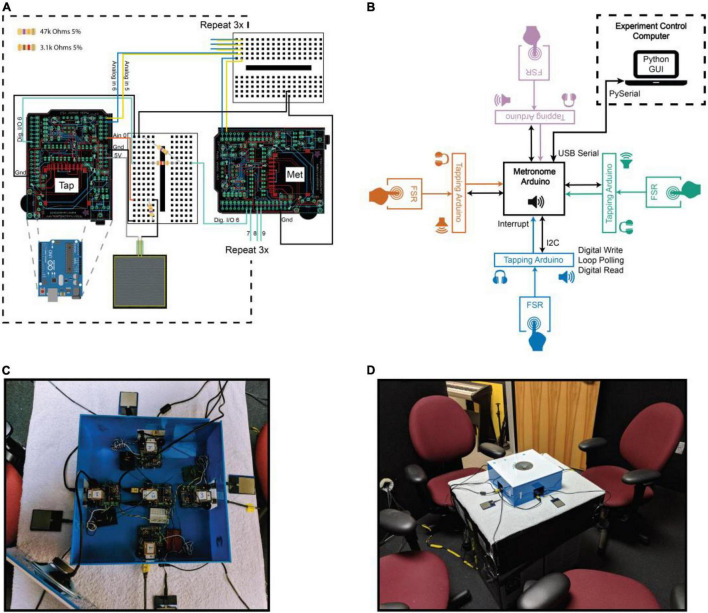
Overview of the GEM system. **(A)** Wiring diagram illustrating the connections between one tapping device (Tap) and the metronome (Met). Note that we repeat these connections three times (indicated by the dotted black box) to arrive at our 4 Tapper + metronome system. Note also that the connections from all Adafruit Wave Shields (v1.1) depicted here in black carry through to the Arduino Uno boards underneath them to which they are attached, indicated by the dotted gray lines connecting the blue Arduino Uno (bottom left) to the Wave Shield. **(B)** Schematic of all devices comprising the multi-person adaptive metronome and their means of communication. The metronome Arduino can be in either an “idle” or “run” state. In the idle state, the Experimental Control Computer (ECC; housed in a separate experiment control room, represented by dotted black box) can set experiment parameters on the metronome Arduino, which in turn sets parameters on the tapping Arduinos. Upon receiving the message from the ECC to start a trial, the metronome Arduino is in a constant loop of producing metronome tones according to our adaptive algorithm, registering participant taps, and sending data packets to the ECC. The tapping Arduinos are constantly polling, reading the value of the FSR input pin. When the FSR pin exceeds a specified threshold (a tap occurs), a digital output pin is pulsed on, resulting in the triggering of an interrupt on the metronome Arduino. Participants can hear the sound of their own tap through headphones, speakers, or not at all. The metronome is always heard through a speaker. **(C)** A photo of the system used in these experiments. **(D)** The view of our multi-person adaptive metronome, from the participants’ perspective. The speaker in the top center of the box played the tones from the metronome (all Experiments). During experiments in which participants could hear the sound produced by their own tap, they used headphones, which can be seen hanging from the side of the table. During Experiment 5, when participants could hear everyone in the group, we placed an additional speaker to the left of each tap pad.

The hardware and software comprising the multi-person adaptive metronome were custom-built using open-source tools. The hardware consists of five Arduino Uno devices^1^, five Adafruit Wave Shields (v1.1^2^) for playing sound, and four force sensitive resistors (InterlinkElectronics, 2018) for tapping. The Wave Shields are connected to the Arduinos to enable them to produce sound (i.e., the metronome sound and the sound produced by tapping on the tap pads). For each participant, a single force sensitive resistor (FSR) is used as a tap pad and is connected to a single Arduino which registers the taps, communicates with the metronome Arduino, and plays sounds triggered by the participant’s taps. The fifth Arduino is the metronome Arduino. It integrates inputs from the other Arduinos, implements the metronome timing function, generates metronome tones, and transmits event data to a connected computer. All four Arduinos responsible for registering taps are wired into the metronome Arduino (see Figures 1A,B). A speaker is connected to the Wave Shield of each Arduino. The Wave Shields have a headphone jack and volume wheel, allowing the experimenter to choose whether participants should hear sounds produced during the experiment *via* a speaker, individually through headphones, or not at all.

Software associated with the multi-person adaptive metronome is freely available for download. All software was custom built in C++, Python 2.7, and Julia 0.6.0 (Bezanson et al., 2012). Separate programs were compiled for the metronome and tapping Arduinos, and downloaded to the respective Arduinos. Because the adaptive metronome code executed directly on the metronome Arduino, the adaptivity calculations were efficient, with minimal delay for registering taps and adjusting subsequent metronome tones. The metronome Arduino was connected to an experiment control computer (ECC)–a MacBook Pro (Apple, Inc., Cupertino, CA, United States)–*via* USB serial port running at 115,200 baud. The PySerial package was used for two-way communication between the metronome Arduino and the ECC. A custom-written Python program running on the ECC handled the randomization of experiment conditions, transmitted alpha values for each trial to the metronome Arduino, received data from the metronome Arduino that was streamed to custom binary files, and displayed relevant information to the experimenter.

We implemented a graphical user interface (GUI) to allow for easily customizable data collection procedures. With a simple Python script, experimenters can set parameters of the metronome (e.g., tempo, adaptivity percentages, number of repetitions for each adaptivity condition, number of tapping Arduinos in the experiment, data output paths, etc.). From the GUI, experimenters can input participant IDs, control the start and stop of runs or practice runs, and monitor the time remaining in the experiment. Please see Figure 1 for an overview of our hardware, software, and experimental set-up. Also note that all code to create the system is publicly available: https://github.com/janatalab/GEM.

For single person experiments (Exps. 1 and 2), participants always tapped on the same individual tap pad (of the four possible tap pads). At their seat, participants also had an iPad running our experiment web interface, Ensemble (Tomic and Janata, 2007), to answer surveys. All experimental instructions, presentation code, data, and analysis code related to the experiments reported in this paper are available in a separate GitHub respository: https://github.com/janatalab/GEM-Experiments-POC.

#### Adaptivity Calculations

The calculation of tap asynchronies and subsequent metronome adjustments was defined in relation to the metronome period, or inter-onset-interval (IOI). The fundamental temporal unit for registering taps in relation to a metronome tone was defined as +/– half of the metronome IOI. For example, with a metronome IOI of 500 ms, taps registered in the window spanning −250 ms before to +250 ms after the tone, are ascribed to the current tone. When a tap is registered on the tap pads of any of the tapping Arduinos, an interrupt is triggered on the metronome Arduino. With nanosecond precision, interrupts are a precise way to register the timing of an event.

Once the temporal window of registering taps for the current tone has elapsed (e.g., 250 ms after the metronome tone for an IOI of 500 ms), the times of the taps registered for that tone are used to calculate the timing of the next metronome tone. Upon calculating the time of the next metronome tone, and setting a corresponding timer, the data are sent to the ECC with less than 1 ms delay. The data packet sent to the ECC after every metronome event window is 12 bytes: 4 bytes for the time of the metronome tone onset, and 2 bytes for each tapper’s tap time, relative to the metronome (8 bytes total). The ECC streams these data to a custom-formatted binary data file.

#### Personality and Individual Factors

In each experiment, all participants completed the Goldsmith’s Music Sophistication Index (GOLD-MSI; Müllensiefen et al., 2014), the Internality, Powerful Others, and Chance Scales (IPC scales; Levenson, 1981), the Brief form of the Affective Neuroscience Personality Scales (BANPS; Barrett et al., 2013), and a short form assessing basic demographic information. The GOLD-MSI assesses six subcategories of possible musical expertise: *active engagement*, *perceptual abilities*, *musical training*, *singing abilities*, and *emotions*, which, when combined, generate an overall *general musical sophistication* score. We assumed people with higher musical sophistication would exhibit less variable tapping performance. The BANPS is the brief version of Affective Neuroscience Personality Scales (Davis et al., 2003; Davis and Panksepp, 2011) and measures behavioral traits in relation to six primary hypothesized neural affective systems: *play*, *seek*, *care*, *fear*, *anger*, and *sadness*, each of which has hypothesized neural correlates. These scales were included to explore potential relationships between neuromodulatory systems and interpersonal dynamics. The IPC scales (Levenson, 1981) measure the degree to which participants think that: (1) they have control over their own life (*internality*), (2) others control events in their life (*powerful others*), and (3) chance dictates their life (*chance*). Previously, the internality subscale was associated with the degree to which participants were categorized as leaders or follows in a single-person adaptive metronome task (Fairhurst et al., 2014). In the current experiments, we wondered whether those with higher internality score may be more closely synchronized with the metronome and/or the group. Collectively, the above surveys allow for an exploratory analysis of the degree to which person-level factors influence tapping behavior and subjective experience in group settings.

## Experiment 1

Though the multi-person adaptive metronome was built with four tappers in mind, our initial experiments involved only one tapper so we could confirm the system worked as expected in a known scenario. Thus the goal of the first experiment was to replicate the findings of Fairhurst et al. (2013) with our new Arduino-based system.

### Methods

#### Participants

A statistical power analysis was performed for sample size estimation, based on data from Fairhurst et al. (2013) (*N* = 16), comparing metronome adaptivity = 0 to metronome adaptivity = 0.25. The effect size in this original study was −0.95, considered to be large using Cohen’s (1988) criteria. With an alpha = 0.05 and power = 0.90, the projected sample size needed with this effect size for the current experiment was approximately *N* = 12, for this simplest comparison between alpha conditions. We thus sought to collect data from at least 20 participants to allow for attrition. Data collection was set to stop when a maximum of 30 participants had completed the task, or the academic term ended, whichever came first.

Twenty-one undergraduate students from the University of California, Davis, participated in exchange for partial course credit. Data from one participant was discarded because of self-report of abnormal hearing. Data from two participants could not be used due to technical issues with our internet connection during data collection, which resulted in loss of survey data. After the additional data cleaning procedures described below, we had a total of 15 participants, aged 21 +/- 2 years; 8 females. For all experiments reported in this paper, participants provided informed consent in accordance with a protocol approved by the Institutional Review Board of the University of California, Davis.

#### Stimuli

The only sound heard throughout the experiment was the sound of the metronome. This sound was a marimba sample from GarageBand, with pitch A2 and a 400 ms duration, played through a speaker connected to the metronome Arduino at a comfortable listening volume. All stimuli discussed in this paper are available in the GEM-Experiments-POC repository.

#### Procedure

The participant was seated in a sound-attenuating room and instructed to synchronize their tapping with the metronome, starting with the third tone. They were told to try to maintain the initial established tempo of the metronome from the first two tones and that the metronome would adapt based on their performance. Following the delivery of instructions, we ensured participants understood the task and showed them how to tap on the force-sensitive-resistors with the index finger of their dominant hand. Participants then completed one practice round of tapping (∼13 s); a maximum of three practice trials was possible, if participants struggled to understand the task. Metronome adaptivity was always at 0 during the practice rounds.

Throughout the experiment, participants completed ten rounds of tapping at each of five adaptivity levels (0, 0.25, 0.5, 0.75, 1), for a total of 50 rounds of tapping. Adaptivity level was randomized across rounds of tapping. Each round consisted of 25 metronome tones and lasted approximately 13 s. The initial metronome tone inter-onset-interval for all rounds was set to 500 ms (120 beats per minute). Following each round of tapping, participants answered a short questionnaire which assessed, on a set of 5-point scales, the degree to which they felt: (1) synchronized with the metronome, (2) in the groove, (3) they had influence over the metronome pulse, (4) the task was difficult, and (5) they would have liked to continue with the task. We defined synchronization as the degree to which participants thought their taps were aligned with the tones of the metronome (from an objective perspective) and “being in the groove” as an effortless, pleasurable feeling of oneness with the metronome (a subjective experience; see Janata et al., 2012). This distinction was explained to participants during the instruction period (for full instructions text, please visit our GEM-Experiments-POC repository wiki). In total, all rounds of tapping, including post-tapping surveys, typically lasted 30–40 min. Participants then completed the personality-related and demographic surveys, which typically took 15–20 min. The entire experiment lasted approximately 1 h.

### Data Analysis

Binary tapping data files were converted to .csv using a custom file parser in Julia (available in the GEM repository). All data were then preprocessed and concatenated using custom scripts in MATLAB (Mathworks, Natick, MA, United States). Note that while all hardware and software used to build and run the adaptive metronome is open source, MATLAB is not. We used MATLAB purely due to its convenient interface with our web-based data collection tool, Ensemble (Tomic and Janata, 2007). After concatenating all cleaned tapping and survey data into tables in MATLAB, the remaining analyses were conducted in Python. The anonymized data tables for each experiment and the analysis code are available in the GEM-Experiments-POC repository. All statistical analyses can be recreated using the provided data tables and Jupyter notebooks.

#### Tapping Data

Participants who missed 30% or more of the required number of taps were eliminated from further analysis. Of the data remaining, any rounds missing > 30% of the taps were discarded. If these data cleaning procedures resulted in a participant not having any observations for any of the adaptivity conditions, that participant was removed from further analyses (3 participants). All tapping data were analyzed with respect to metronome adaptivity condition. The dependent measure of interest was the standard deviation of tap asynchronies, which serves as a measure of synchronization stability, as in Repp and Keller (2008) and Fairhurst et al. (2013).

#### Survey Data

All questionnaires were analyzed *via* a custom MATLAB implementation of the original instrument authors’ scoring metrics. For the post-tapping survey, data were z-score normalized within each rating scale for each participant. Correlations among surveyed variables were checked and an exploratory factor analysis was conducted.

#### Statistical Analyses

All statistical analyses were conducted in Python using the *pingouin* (Vallat, 2018), *statmodels* (Seabold and Perktold, 2010) and *factor-analyzer* (Biggs, 2020) packages. All code is available in the associated Jupyter notebooks.

### Results

**FIGURE 2:**
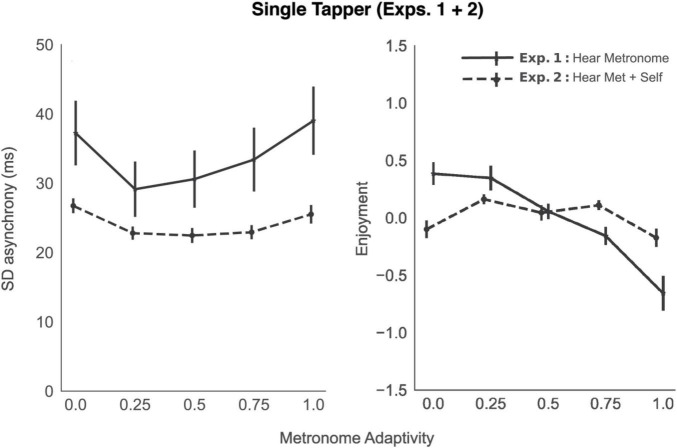
Tapping performance (standard deviation of tap—metronome asynchrony), left panel, and subjective enjoyment, right panel, averaged across participants, as a function of metronome adaptivity, in the two single tapper experiments. Solid black lines represent experiments in which participants could only hear the sound produced by the metronome; dashed lines, experiments in which participants could hear the metronome and the sound of their own tap. Error bars represent standard error of the mean.

The tapping results for Experiment 1 are plotted in Figure 2, left panel (solid line), as a function of metronome adaptivity condition (α). A repeated measures analysis of variance (ANOVA) revealed a main effect of α condition on tapping SD asynchrony, F(4, 56) = 8.16, *p* = 0.002, η^2^ = 0.26. Paired *t*-tests were calculated one-sided, based on a level of alpha = 0.05. The *t*-tests revealed significant differences in SD asynchrony between the baseline condition (α = 0) and what we might call optimally adaptive conditions: α = 0.25, t(14) = 2.81, *p* = 0.007, *d* = 0.48, Δ async = −8.10 ms, and α = 0.5, t(14) = 2.92, *p* = 0.006, *d* = 0.39, Δ async = −6.64 ms. There were no significant differences from the baseline condition when α = 0.75 or 1, both *t* < 1.

Turning to subjective ratings, we found participants’ perceived synchrony with the metronome was significantly negatively correlated with their tapping SD asynchrony [*r*_*rm*_(603) = −0.40, 95% CI (−0.47, −0.33), *p* < 0.001]. Reduced asynchronies (better synchrony with metronome) led to a greater sense of subjective synchrony, suggesting participants have at least a moderately accurate assessment of their own tapping performance. Before analyzing subjective ratings in relation to metronome adaptivity, we checked for correlations among the individual items and found all showed significant correlations with each other (see Supplementary Appendix 1). Thus, we performed an exploratory factor analysis to identify one or more latent variables. Two preliminary tests confirmed the suitability of applying a factor analysis to these data (Bartlett’s test of sphericity = 810.04, *p* < 0.001; Kaiser-Meyer-Olkin measure of sampling adequacy = 0.738, *p* < 0.001). Eigenvalues and a scree plot indicated a 1-factor solution. We therefore ran the factor analysis again, specifying a one factor solution, using the maximum likelihood method, and no rotation (rotation is not possible with one factor). Factor loadings and communalities are shown in Table 2. Groove, synchrony, and liking all loaded strongly and positively onto this factor, while difficulty had a high negative loading. With a loading less than 0.3, participants’ felt influence over the pulse played only a small role in this overall factor and was therefore excluded from the overall factor score. We hereafter refer to this factor as “Enjoyment.”

**TABLE 2:** Item loadings for the Enjoyment factor (Exp. 1).

Item	Enjoyment	Communality
Groove	0.663	0.450
Synchrony	0.836	0.699
Liking	0.460	0.212
Difficulty	−0.772	0.596

*Cumulatively, the single factor enjoyment had a sum of squared loadings of 2.01, explaining a proportional and cumulative 49% of variance.*

Participants’ enjoyment factor scores are plotted as a function of metronome adaptivity in Figure 2, right panel (solid line). A repeated measures ANOVA revealed a main effect of α condition on enjoyment, F(4, 56) = 13.65, *p* < 0.001, η^2^ = 0.49. There was no significant difference between baseline (α = 0) and α = 0.25. However, a significant decrease in enjoyment was observed between baseline and α = 0.5, 0.75, and 1. Detailed statistics for all pairwise comparisons can be viewed in the associated Jupyter Notebook.

### Discussion

The tapping results of Experiment 1 replicate those of Fairhurst et al. (2013), using our new Arduino-based adaptive metronome. The Arduino-based system functions as expected: it brought participants into greater synchrony with the metronome at adaptivity levels of 25–50% (compared to baseline), as in Fairhurst et al. (2013). At higher levels of adaptivity (75% or 100%) participants’ tapping performance returned to baseline or worse. We can, therefore, conclude, like Fairhurst et al. (2013), that it is possible for the metronome to be in “optimal” vs. “overly” adaptive states, as could be the case with a real person with whom one might interact. By simulating such states in a controlled way, we can assess the degree to which optimal vs. extreme adaptivity influence participants’ experience.

With respect to subjective experience during the task, we firstly found participants’ perceived synchrony with the metronome was correlated with their objective (measured) tapping synchrony. We also found their synchrony rating is highly correlated with their groove, liking, and difficulty ratings. An exploratory factor analysis showed all of these ratings could be reduced to one collective factor which we labeled Enjoyment. These correlations between our rating items echo previous studies that have also often found associations between groove, liking, and difficulty (e.g., Janata et al., 2012; Hurley et al., 2014; Witek et al., 2014).

In examining participants’ enjoyment scores in relation to metronome adaptivity, we did not see any increase in enjoyment with optimal adaptivity, though we did find significant decreases in enjoyment when the metronome adapted by 50% or more. It is hard to directly compare these subjective findings with those of Fairhurst et al. (2013), as they used only difficulty, influence, and synchrony ratings on a visual analog scale, ranging from 0 to 10, whereas we included additional items, all on 5 pt. scales. Nonetheless, our results follow their overall pattern of subjective rating findings (see their Supplementary Table 1), with about the same ratings observed in baseline and 25% adaptivity conditions, followed by an increased difficulty (in our case, decreased enjoyment), at adaptivity levels 50% and higher, compared to baseline. Whether the overall patterns in tapping synchrony and subjective experience reported here will remain the same when participants receive auditory feedback about their taps is the topic of Experiment 2.

## Experiment 2

Before applying our adaptive metronome to group contexts, we first wanted to more clearly characterize its functioning in the single-person use case. To this end, we repeated Experiment 1, but additionally provided participants auditory feedback from their taps. We hypothesized such feedback would lead to better synchronization with the metronome and increased enjoyment of the task.

### Methods

All methods were identical to those of Experiment 1, with the following exception: participants could hear the sound produced by their own tap, through headphones. This sound was a woodblock sample from the Proteus 2000 sound module. Participants could still hear the metronome through its speaker. Therefore, before the experiment started, we had participants calibrate their headphone volume such that the volume of their tap was perceptually matched to that of the metronome. Twenty-eight participants took part in the study. Two were removed according to the data cleaning procedures outlined in Exp. 1, for a total of 26 remaining participants, mean age 20 +/– 2 years; 24 were female. No participant participated in more than one experiment reported in this paper.

### Results

The tapping data for Exp. 2 are plotted in Figure 2, left panel (dashed line). A repeated measures analysis of variance again showed a main effect of α condition on tapping SD asynchrony Exp. 2: F(4, 100) = 8.69, *p* < 0.001, η^2^ = 0.26. Comparing means, we not only found significant differences between baseline and α = 0.25, t(25) = 5.33, *p* < 0.001, *d* = 0.77, Δ async = −3.94 ms, and α = 0.5, t(25) = 4.39, *p* < 0.001, *d* = 0.78, Δ async = −4.28 ms, but also between baseline and α = 0.75, t(25) = 4.51, *p* < 0.001, *d* = 0.72, Δ async = −3.81 ms.

To assess the effect of auditory feedback on tapping SD asynchrony, we conducted a mixed ANOVA with auditory feedback as a between subjects predictor, adaptivity condition as a within subjects predictor, and their interaction. Compared to Exp. 1, we found tapping performance improved significantly when participants receive auditory feedback from their taps [main effect of auditory feedback, F(1,39) = 8.57, *p* < 0.001, η^2^ = 0.18]; on average, participants’ SD async decreased by 9.79 ms (i.e., became more stable). The main effect of adaptivity was also significant [F(4,156) = 10.86, *p* < 0.001, η^2^ = 0.22], as was the interaction between adaptivity and auditory feedback [F(4,156) = 2.59, *p* < 0.001, η^2^ = 0.06]—at higher levels of adaptivity, the effect of auditory feedback on tapping performance was more pronounced.

With regard to subjective experience, participants’ perceived synchrony with the metronome was significantly negatively correlated with their tapping SD asynchrony [*r*_*rm*_(1,085) = −0.30, 95% CI (−0.36, −0.25), *p <* 0.001], suggesting accurate assessment of their own tapping capabilities. As in Experiment 1, we again found significant correlations between subjective rating scales (see Supplementary Appendix 1), and, therefore, conducted a confirmatory factor analysis, in line with the exploratory results of Exp. 1. Two preliminary tests confirmed the suitability of this approach (Bartlett χ^2^= 1,307.07, *p* < 0.001; KMO = 0.727, *p* < 0.001). Our 1 factor solution showed all loadings in the same directions and relative magnitudes as in Exp. 1, Table 2 (see Supplementary Appendix 2 for the loadings of this experiment). We therefore continued with the labeling of this factor as Enjoyment.

Participants’ Enjoyment factor scores are plotted in Figure 2, right panel (dashed line). A repeated measures ANOVA revealed a main effect of α condition on enjoyment score, F(4, 100) = 4.04, *p* = 0.015, η^2^ = 0.14. All paired, *t*-tests were calculated one-sided, based on a level of alpha = 0.05. Compared to baseline (α = 0), there was a significant increase in enjoyment at α = 0.25 [t(25) = −3.30, *p* = 0.014] and α = 0.75 (*t* = −2.03, *p* = 0.03).

To assess the effect of auditory feedback on enjoyment, we conducted a mixed ANOVA with auditory feedback as a between subjects predictor, adaptivity condition as a within subjects predictor, and their interaction. While the main effect of auditory feedback was not significant, the interaction between auditory feedback and adaptivity condition was [F(4,156) = 8.84, *p* < 0.001]. With no adaptivity (baseline condition), having auditory feedback was significantly less enjoyable [t(30) = 3.91, *p* = 0.001], whereas at higher levels of adaptivity (α = 0.75 and 1), auditory feedback resulted in greater enjoyment. All comparisons of means can be found in the associated Jupyter Notebook.

### Discussion

When participants received auditory feedback from their taps, they achieved greater synchrony with the metronome (in all conditions). In terms of metronome adaptivity, performance was improved, even at higher levels of adaptivity (75%), but returned to near baseline with 100% adaptivity. Auditory feedback resulted in a significant overall decrease in SD asynchrony, compared to no feedback (overall difference in means between Exp. 1 and 2 was ∼10 ms). Though there was no overall difference in enjoyment scores between Exps. 1 and 2, in Experiment 2, we did find a significant improvement in participants’ enjoyment at 25% and 75% adaptivity, compared to no adaptivity baseline, as well as an interaction between auditory feedback and alpha condition. Whereas in Experiment 1, enjoyment plummeted at higher levels of adaptivity, in Experiment 2, when participants received auditory feedback, enjoyment remained high. These results speak to the relevance of auditory feedback in influencing both tapping accuracy and subjective enjoyment in an adaptive metronome context. We thus continued to explore the effect of auditory feedback in group tapping contexts, reported below.

## Experiment 3

After replicating and extending single-person adaptive metronome studies using our Arduino-based system, we next sought to validate the use of an adaptive metronome in multi-person contexts.

### Methods

#### Participants

Because the power analysis detailed in Experiment 1 was based on individuals rather than groups, and no previous adaptive metronome group-tapping experiment existed to use for an effect size calculation, we erred on the side of more groups, with data collection set to stop when a maximum of 35 groups had completed the task, or the academic term ended, whichever came first. Participants were not systematically assigned into specific groups. They could register for a timeslot in the experiment *via* UC Davis’s online recruitment system. Hence, the four people who had signed up (for, e.g., the 9 am–10 am timeslot) were all grouped together. One hundred twenty-four undergraduate students (31 groups of 4) from the University of California, Davis, participated in exchange for partial course credit. Four groups had to be removed due to technical difficulties (Wi-Fi issues during survey completion). The remaining 108 participants (27 groups) had a mean age of 21 +/- 3 years; 83 were female.

#### Stimuli

Same as in Experiment 1. Participants only heard the metronome (marimba sample) and did not hear any sound produced by their own or others’ taps. As in Experiment 1, the initial metronome tone inter-onset-interval for all rounds was set to 500 ms (120 beats per minute).

#### Procedure

The procedure was largely the same as in Experiment 1, with the following exceptions. Participants were instructed to keep their gaze on their own finger as they tapped and not to speak with the other participants during the experiment. Surveys, such as the IPC scales, were taken before the tapping experiment started, rather than at the end, so as not to be influenced by any perceived social dynamics during the experiment. The post-tapping survey presented after each round asked an additional question about how in synchrony participants felt with the others. Participants completed six rounds of tapping at each of four adaptivity levels (0, 0.35, 0.7, 1), for a total of 24 rounds of tapping. Each round lasted approximately 30 s (as opposed to 13 s in Experiments 1 and 2). As previously, adaptivity level was randomized across rounds of tapping. Instructions remained the same; we acknowledge that there is perhaps an ambiguity in how participants could now interpret the phrase “based on your performance” (either as an individual or as a group)—both would be acceptable and accurate interpretations.

### Data Analysis

All data concatenation, cleaning, and analysis procedures were identical to those for Exps. 1 and 2, with the following exceptions.

#### Tapping Data

Groups in which 30% or more of the required number of taps were missed (across the entire group) were eliminated from further analysis.

The main dependent measure of interest was the standard deviation of the mean asynchrony of the group, relative to the metronome tones (referred to below as SD async). An additional metric of interest was the relative performance of the individuals with respect to the group, which we calculated as the individual’s SD asynchrony with respect to the metronome minus the group’s SD asynchrony with respect to the metronome, as defined in the following sequence of equations. Equation 3 represents the tapping asynchrony of the individual (*i*) with respect to the metronome (*met*) for each metronome window (*w*),


(3) asyncwi=ttapwi-tmetw

Equation 4 the asynchrony of the group (*G*) with respect to the metronome for each metronome window,


(4) asyncwG=1I∑i=1Ittapwi-tmetw

and Equation 5 the difference in standard deviation of tapping asynchrony between the individual and the group.


(5) $$SDasync_{differencei}=\sqrt{\frac{{\sum \left(async_{wi}-{\text{μ}}_{{\text{async}}_{\text{i}}}\right)}^{2}}{w}}-\sqrt{\frac{{\sum \left(async_{wG}-{\text{μ}}_{{\text{async}}_{G}}\right)}^{2}}{w}}$$

We were also interested in the stability of the group members’ tapping relative to each other and calculated this by first taking the standard deviation of the four participants’ asynchronies for each metronome window:


(6) SDGw=∑(asyncwGi - μasyncwG)2I  

Then taking the standard deviation of those standard deviations over time:


(7) SDSDG=∑(SDGw -μSDG)2W

**FIGURE 3:**
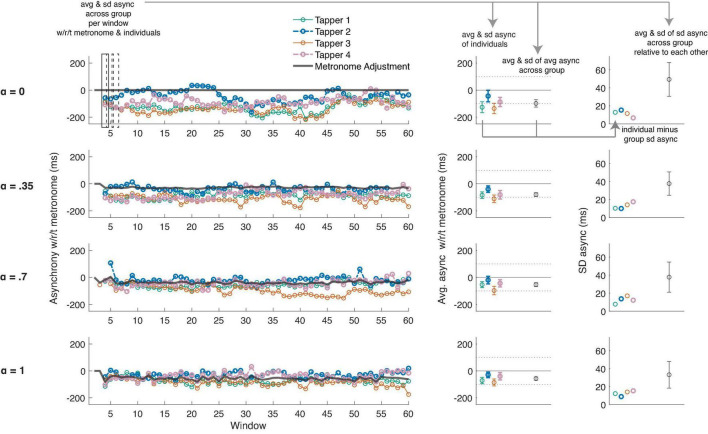
An example run (left panels) at each adaptivity condition (rows) during the group tapping experiments. The metrics illustrated in this figure are applicable to all group tapping experiments, though this particular example comes from Exp. 5 (when all participants could hear each other and the metronome). The individual, colored lines and dots represent the individual tappers in the group. The solid gray line represents the adjustments of the metronome. Note that there are no adjustments in the top left panel when α = 0 (i.e., the control condition that mimics a standard metronome). In the middle panels, the average asynchrony (dots) and SD of the average asynchrony (error bars) of all individuals (colored dots) and the group (black dot), with respect to the metronome, are plotted. A solid line at 0 and dotted lines +/– 100 ms are plotted for reference. The group SD asynchrony (error bars of black dot) was our main metric of interest (see Figure 4). In the right-most panels, the colored dots show the difference between each individual’s SD asynchrony and the group SD asynchrony. This metric indexes how variable the individual is vs. the group as a whole, with respect to the metronome, and is used in our models assessing individual differences. The black dot represents the average of the SD asynchrony of the individuals, across all windows; the error bar is the SD of this average SD asynchrony, which we consider an index of how variable individuals’ taps are with respect to each other (rather than the metronome).

These different metrics are illustrated in Figure 3 and detailed in its caption.

#### Survey Data

All questionnaires were analyzed *via* a custom MATLAB implementation of the original instrument authors’ scoring metrics. For the post-tapping survey, data were z-score normalized within each participant, each rating scale, then averaged across participants within each group.

### Results

**FIGURE 4:**
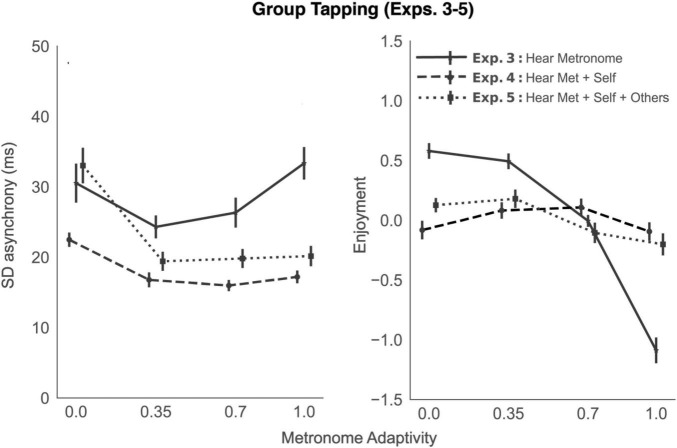
Tapping performance and subjective enjoyment, averaged across participants, as a function of metronome adaptivity, in the three group tapping experiments. Solid black lines represent experiments in which participants could only hear the sound produced by the metronome; dashed lines, experiments in which participants could hear the metronome and the sound of their own tap; dotted lines, experiments in which participants could hear the metronome, themselves, and all others in the group. Error bars represent standard error of the mean.

**FIGURE 5:**
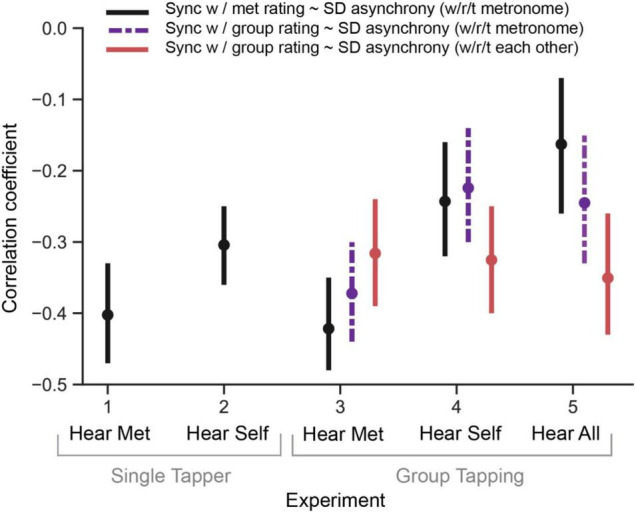
Correlations between motor tapping asynchrony and subjectively rated synchrony with the metronome in all experiments. Note that in group tapping experiments, we have additional data about how in synchrony participants felt with the group. We analyzed that data separately as a function of synchrony with respect to the metronome (dotted purple line) and synchrony with respect to everyone in the group (red line). Error bars represent confidence intervals of the correlation coefficients.

A repeated measures ANOVA showed a main effect of α condition on the average group tapping SD asynchrony [F(3, 78) = 11.33, *p* < 0.001, η^2^ = 0.30]; see Figure 4, left panel, solid line. Comparisons between all means showed significant improvements in tapping performance at α = 0.35 [t(26) = 3.02, *p* < 0.001, *d* = 0.54, Δ async = −6.22 ms], and α = 0.7 [t(26) = 2.09, *p* < 0.001, *d* = 0.33, Δ async = −4.19 ms], but not at α = 1, compared to baseline. The group’s tapping SD asynchrony was negatively correlated with both the average (across the group) of each individuals’ perceived synchrony with the metronome [*r*_*rm*_ (605) = −0.42, 95% CI (−0.48, −0.35), *p* < 0.001] and the average (across the group) of each individuals’ perceived synchrony with the group [*r*_*rm*_ (605) = −0.37, 95% CI (−0.44, −0.30), *p* < 0.001]. Collectively, these correlations indicate groups can accurately judge their own tapping stability. Similarly, the group’s tapping variability with respect to each other (i.e., SD of SD async, Figure 3, right panel, black error bars) was significantly correlated with the average perceived synchrony of the group [*r*_*rm*_ (605) = −0.32, 95% CI (−0.39, −0.24), *p* < 0.001]; see Figure 5.

As in the previous experiments, we again found significant correlations between the subjective rating scales (see Supplementary Appendix 1), and, therefore, conducted a confirmatory factor analysis, in line with the exploratory and confirmatory results of Exps. 1 and 2, respectively. Two preliminary tests confirmed the suitability of this approach (Bartlett χ^2^= 2,129.74, *p* < 0.001; KMO = 0.865, *p* < 0.001). Our 1 factor solution showed all loadings in the same directions and relative magnitudes as in Exps. 1 and 2. The additional item present in the group experiments (“To what extent did you feel in synchrony with the other players in the group?”) had a high positive loading, similar to the rated synchrony of the self with the metronome. Factor loadings are shown in Table 3. The Enjoyment factor explains 65% of the variance in the ratings data.

**TABLE 3:** Item loadings for the Enjoyment factor (Exp. 3).

Item	Enjoyment	Communality
Groove	0.893	0.798
Synchrony (w/metronome)	0.937	0.877
Synchrony (w/group)	0.823	0.677
Liking	0.517	0.268
Difficulty	−0.792	0.628

*Cumulatively, the single factor enjoyment had a sum of squared loadings of 3.25, explaining a proportional and cumulative 65% of variance.*

The group’s average Enjoyment factor scores are plotted in Figure 4, right panel (solid line). A repeated measures ANOVA revealed a main effect of α condition on enjoyment score, F(3, 78) = 78.03, *p* < 0.001, η^2^ = 0.75. All paired *t*-tests were calculated one-sided, based on a level of alpha = 0.05. Compared to baseline (α = 0), there was no change in Enjoyment at α = 0.35; however, there was a significant decrease in enjoyment at higher adaptivity levels: α = 0.7 [t(26) = 6.38, *p* < 0.001] and α = 1 (*t* = 10.16, *p* < 0.001).

### Discussion

With this experiment, we show that an adaptive metronome can be employed successfully in a group context. By adapting to the average asynchrony of the group, we find groups of tappers can be brought into greater synchrony (compared to baseline). Alternatively, too much adaptivity results in performance at or worse than baseline. Thus, the tapping stability of groups can be manipulated using our multi-person adaptive metronome in a manner analogous to the results observed in single tapper contexts without auditory feedback (Exp. 1).

In terms of subjective experience, we replicate the factor structure of our previous experiments, with the addition of feeling in synchrony with the group also loading onto the Enjoyment factor. We found enjoyment significantly decreased at higher levels of adaptivity but not at optimal adaptivity (35%), mimicking the effects observed in Exp. 1. We also found that groups are generally able to accurately assess the stability of their own tapping performance, as indicated by the significant correlation between their ratings of synchrony and their measured tapping SD asynchrony, on the trial level. In the following experiments, we explore the effect of auditory feedback on group tapping stability and enjoyment.

## Experiment 4

### Methods

#### Participants

One hundred and four undergraduate students (26 groups of 4) participated. One group had to be removed due to technical difficulties during data collection. In total there were 25 groups, 100 participants, with a mean age of 20 +/– 2 years, 79 female.

#### Stimuli

Participants heard the metronome (marimba sample) through its speaker and their own tap sound through headphones. All participants’ taps produced the same sound (the woodblock sample from Experiment 2). Before the experiment started, we had participants calibrate their headphone volume such that the volume of their tap was perceptually matched to that of the metronome.

#### Procedure

Same as Exp. 3.

### Results

A repeated measures ANOVA showed a main effect of α condition on the average group tapping SD asynchrony [F(3, 72) = 22.09, *p* < 0.001, η^2^ = 0.48]. Comparisons between all means showed significant differences between α = 0 and all other conditions. Tapping performance significantly improved at α0.35 [t(24) = 5.17, *p* < 0.001, *d* = 1.10, Δ async = −5.70 ms], α0.7[t(24) = 7.71, *p* < 0.001, *d* = 1.43, Δ async = −6.51 ms], and α 1[t(24) = 4.51, *p* < 0.001, *d* = 1.10, Δ async = −5.28 ms]. In brief, tapping performance remained significantly improved, no matter how adaptive the metronome was (see Figure 4, left panel, dashed line).

As in Experiment 3, the group’s tapping SD asynchrony was negatively correlated with the average perceived synchrony with the metronome [*r*_*rm*_ (541) = −0.24, 95% CI (−0.32, −0.16), *p* < 0.001] and the average perceived synchrony with the group [*r*_*rm*_ (541) = −0.22, 95% CI (−0.30, −0.14), *p* < 0.001]. Similarly, the group’s tapping variability with respect to each other was significantly negatively correlated with the average perceived synchrony of the group [*r*_*rm*_ (541) = −0.33, 95% CI (−0.4, −0.25), *p* < 0.001].

Ratings data were again subject to a confirmatory factor analysis (see Factor Loadings in Supplementary Appendix 2). The group’s average Enjoyment factor scores are plotted in Figure 4, right panel (dashed line). A repeated measures ANOVA showed no main effect of α condition on enjoyment score, F(3, 72) = 1.53, *p* = 0.213, η^2^ = 0.06.

### Discussion

We successfully replicated the effect observed in Experiment 3: The multi-person adaptive metronome can be used to bring groups of tappers into greater synchrony, compared to their baseline. In contrast to Exp. 3, in the current experiment with self-related auditory feedback, we found groups were able to maintain enhanced synchrony, even at higher levels of adaptivity. These results are interesting as they suggest 1) groups are able to more flexibly adapt to a greater degree of difficulty when they have additional information (auditory feedback), and/or 2) individuals in groups are able to synchronize better with the metronome and thereby each other when they receive feedback, so as to negate the potentially disruptive effects of a highly adaptive metronome. Pushing the group into a difficult and unpleasant state may, therefore, require using adaptivity levels greater than 100%. Future experiments should explore this possibility. In Exp. 5, we continued, for consistency, with the same levels of adaptivity as we added auditory feedback about the other tappers in the group.

## Experiment 5

### Methods

#### Participants

One hundred and four undergraduate students (26 groups of 4) participated. Three groups were removed due to technical issues. An additional, three groups were removed during data cleaning (outlined below). In total there were 20 groups, or 80 participants, with a mean age of 21 +/- 4 years, 57 female.

#### Stimuli

Participants heard the metronome (marimba sample) through its speaker and their own taps (woodblock sample) through their own individual speakers. In other words, all participants’ taps produced the same sound, and all participants could hear the tap sounds produced by all others.

#### Procedure

Same as Exp. 3.

### Results

A repeated measures ANOVA showed a main effect of α condition on the group tapping SD asynchrony [F(3, 54) = 33.73, *p* < 0.001, η^2^ = 0.65]; see Figure 4 (left panel, dotted line). As in Experiment 4, tapping performance improved significantly in all adaptivity conditions, compared to baseline [α0.35:t(18) = 7.29, *p* < 0.001, *d* = 1.61, Δ async = −13.61 ms; α0.7:t(18) = 6.19, *p* < 0.001, *d* = 1.56, Δ async = −13.21 ms; α1:t(18) = 6.23, *p* < 0.001, *d* = 1.49, Δ async = −12.86 ms].

As in Experiments 3 and 4, the group’s tapping SD asynchrony was negatively correlated with the average perceived synchrony with the metronome [*r*_*rm*_ (407) = −0.16, 95% CI (−0.26, −0.07), *p* < 0.001] and the average perceived synchrony with the group [*r*_*rm*_ (407) = −0.25, 95% CI (−0.33, −0.15), *p* < 0.001]. The group’s tapping variability with respect to each other was significantly negatively correlated with the average perceived synchrony of the group [*r*_*rm*_ (407) = −0.35, 95% CI (−0.43, −0.26), *p* < 0.001].

A comparison of the correlation coefficients among experiments is shown in Figure 5. Across all experiments, it is interesting that the strongest correlations between subjective feelings of synchrony and actual measured SD synchrony occur with no auditory feedback (i.e., participants most accurately judge their tapping performance when they do not have auditory feedback, despite their being worse at the task). It is also interesting that the SD asynchrony of group members with respect to each other does not seem to change as a function of auditory feedback, meaning in all group experiments participants can equally tell how in sync they are with each other, regardless of auditory feedback (even though their ability to judge their group synchrony with the metronome is best with less auditory feedback).

To examine the effect of auditory feedback on SD asynchrony (across Exps. 3, 4, and 5), we ran a mixed ANOVA, with auditory feedback as a between-subjects factor, adaptivity condition as a within-subjects factor, and their interaction. There was a main effect of auditory feedback in predicting tapping performance [F(2, 281) = 32.29, *p* < 0.001, η^2^ = 0.19]. Compared to no feedback, tapping SD asynchrony decreased on average by 10.52 ms with self-feedback [t(149.96) = 8.23, *p* < 0.001, *d* = 1.11] and by 5.53 with self + other feedback: t(179.97) = 3.46, *p* < 0.001, *d* = 0.5). Transitively, tapping performance was better, on average, in Experiment 4 compared to 5, by 4.99 ms [t(111.19) = 4.14, *p* < 0.001, *d* = 0.68]. In other words, tapping performance is best in Exp. 4, when participants hear the metronome and only themselves, followed by Exp. 5 when all participants can hear each other. Group tapping performance is worst when participants receive no auditory feedback (Exp. 3). There was also a main effect of adaptivity condition [F(3,204) = 38.12, *p* < 0.001, η^2^ = 0.36], as well as an interaction between auditory feedback and adaptivity condition [F(6,204) = 10.75, *p* < 0.001, η^2^ = 0.24]. As is visible in Figure 4, at higher levels of adaptivity, having no auditory feedback significantly worsens performance.

With respect to subjective experience, ratings data affirmed the same factor structure as previous experiments (see Factor Loadings in Supplementary Appendix 2). The group’s average Enjoyment factor scores are plotted in Figure 4 (right panel, dotted line). A repeated measures ANOVA showed a main effect of α condition on enjoyment score, F(3, 54) = 3.96, *p* = 0.013, η^2^ = 0.18. Enjoyment showed no significant difference between baseline and α = 0.35, but did significantly decrease at higher levels of adaptivity (α = 0.7 and 1). In comparing Enjoyment scores across all three group tapping experiments, *via* mixed ANOVA with auditory feedback as a between-subjects factor, adaptivity condition as a within subjects factor, and their interaction, we find a main effect of adaptivity condition [F(6,204) = 21.58, *p* < 0.001, η^2^ = 0.39] but no main effect of auditory feedback. However, we do find a significant interaction between adaptivity and auditory feedback [F(3,204) = 47.55, *p* < 0.001, η^2^ = 0.41]. At baseline (no adaptivity), enjoyment is highest when participants receive no auditory feedback. This effect is reversed at higher levels of adaptivity, where hearing the metronome is significantly more enjoyable than no feedback. Statistics for all *t*-tests can be found in the associated Jupyter notebook.

### Discussion

With this experiment, we again successfully replicated our ability to enhance group synchrony with the metronome by adapting its timing. Contrary to our hypothesis that groups would be even better at tapping when they received auditory feedback about everyone in the group, we found, though hearing everyone resulted in better performance than hearing only the metronome, groups were best in Exp. 4 when they could hear only themselves. Similarly, their enjoyment did not change as much as it did in Exp. 4. In fact, in this experiment, enjoyment did not increase (as in Exp. 4 at optimal levels of adaptivity), but rather decreased at higher levels of adaptivity (70 and 100%), a result more similar to Exp. 3. Implications of these results will be elaborated further in the General Discussion. First, we explore the role of individual differences in shaping tapping performance and subjective experience.

## Individual Differences

### Musical Sophistication

Across all experiments, we were interested in whether participants’ benefit from metronome adaptivity (in terms of improved tapping performance) could be predicted by their musical sophistication. We defined adaptivity benefit as the difference in tapping performance between the baseline and optimally adaptive condition (α = 0.25 and 0.35 for individual and group experiments, respectively). Thus, a positive value corresponds to improved tapping synchrony or enjoyment, while a negative value indicates decreased synchrony or enjoyment. As we found the subscales of the Gold-MSI were all significantly highly correlated with each other, we used only the overall general sophistication index. We felt this general index was better than choosing one subscale, as the musical training, perceptual abilities, and even singing abilities subscales all contain items that would have a direct bearing on tapping abilities.

**FIGURE 6:**
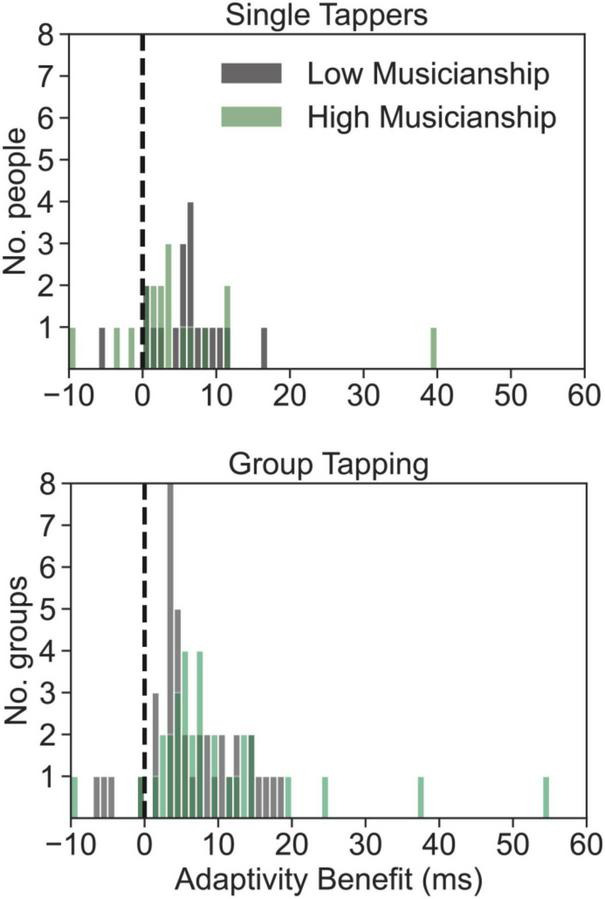
The enhanced tapping accuracy (in ms) that participants experienced during optimally adaptive metronome conditions, as a function of their musical sophistication, in single and group tapping experiments. Participants (top panel) or groups (bottom panel) scoring in the top half of musical sophistication (Gold-MSI) are plotted in green, while those in the bottom half (low musical sophistication) are plotted in gray. Positive values indicate the extent to which participants’ SD asynchrony improved. Negative values indicate the opposite (that participants or group became worse at optimal adaptivity).

To test whether musical sophistication mattered in terms of how much people, or groups, benefitted from metronome adaptivity, we split both the single tapper and group tapping data into two musicianship groups *via* median split (single tapper: low musicianship: *n* = 19, sophistication mean = 57+/– 8; high musicianship: *n* = 18, soph mean = 89 +/– 10; group tapping: low musicianship: *n* = 39, soph mean = 66 +/– 5; high musicianship: *n* = 32, soph mean = 77 +/– 5. The adaptivity benefit of each of the musicianship groups in the single and group tapping contexts was compared against zero, using paired, one-sample, one-tailed *t*-tests. All musicianship groups showed a significant benefit from metronome adaptivity [single / low: t(18) = 5.33, *p* < 0.001, *d* = 1.12, mean benefit = 5.93 ms; single / high: t(17) = 2.05, *p* = 0.028, *d* = 0.48, benefit = 4.89 ms; group / low: t(38) = 6.68, *p* < 0.001, *d* = 1.07, benefit = 6.49 ms; group / high: t(31) = 4.85, *p* < 0.001, *d* = 0.86, benefit = 9.87 ms]; see Figure 6. These results indicate people of all musical levels benefit from an adaptive metronome. There were no significant differences between the means of low vs. high musicianship individuals, or groups, both *t* < 1.5.

### Personality Factors and Individual-Group Asynchronies

While we had no *a priori* hypotheses about which individual traits, besides musical sophistication, and perhaps internality (Fairhurst et al., 2014), might be relevant in the context of group tapping, we were nonetheless interested in exploring predictors of participants’ SD asynchrony with respect to the group, as well as participants’ subjective ratings, with respect to their individual asynchronies.

To start, we asked which person-level variables predict participants’ individual SD asynchrony difference with respect to the group (i.e., the individual minus group SD async; see colored dots in Figure 3, rightmost panels). We ran a linear mixed effects model (*lme4* package in R, Bates et al., 2015; *sjPlot* package, Lüdecke, 2021) with the individual minus group SD asynchrony difference as the dependent variable; all person-level variables were included as fixed effects, while a term specifying adaptivity condition nested within participant, nested in group, nested in experiment was included as a random effect. Note that, as opposed to earlier analyses in which we were interested in adaptivity as a predictor of synchrony, here we wanted to understand the role of person-level features over all conditions. The variance inflation factor for all fixed effects was checked and all were found to be safely < 2.

All model output is presented in Table 4. We found musical sophistication was a significant predictor of individuals’ difference scores. Interestingly, we also found a significant effect for the *sadness* scale of the BANPS (Barrett et al., 2013). Overall, the variance explained by the fixed effects, though significant, is nonetheless small (∼3%), while the random effects (adaptivity, participants, groups, experiment) account for much more variance (∼48%) in the tapping data.

**TABLE 4:** Person-level predictors of individuals’ SD asynchrony differences from the group.

Predictors	Estimates	CI	*p*
(Intercept)	25.50	15.35–35.64	< 0.001
General sophistication (Gold-MSI)	−0.19	−0.24–−0.14	< 0.001
Internality (IPC)	0.14	−0.02–0.31	0.085
Powerful Others (IPC)	−0.01	−0.14–0.12	0.882
Chance (IPC)	0.07	−0.06–0.19	0.309
Play (BANPS)	−0.39	−2.05–1.27	0.644
Seek (BANPS)	−0.39	−1.90–1.11	0.610
Care (BANPS)	−0.07	−1.37–1.22	0.911
Fear (BANPS)	−0.77	−2.11–0.58	0.263
Anger (BANPS)	−0.35	−1.46–0.75	0.533
Sadness (BANPS)	2.22	0.90–3.53	0.001
**Random effects**			
σ^2^	184.74		
τ_00 *alpha:subID:group:exp*_	158.22		
ICC	0.46		
N _alpha_	4		
N _subID_	284		
N _group_	71		
N _exp_	3		
Observations	6508		
Marginal R^2^ / Conditional R^2^	0.031 / 0.478		

*The survey instrument from which each predictor comes is indicated next to each item in parentheses.*

The sadness scale is known to be related to *neuroticism* from the five-factor model, as well as the *behavioral inhibition system* from reinforcement sensitivity theory (Barrett et al., 2013). Behaviors associated with the *sadness* system are often related to loss and grieving, separation distress, or breaking of social bonds; these behaviors are in a somewhat antagonist relationship with the *play* system and its related behaviors. More concretely, higher *sadness* scores are associated with social phobia and negative affect, and show a negative relationship with self-esteem (Barrett et al., 2013). Electrical brain stimulation studies have implicated areas from dorsal periaqueductal gray to anterior cingulate, and the role of neurotransmitters associated with social bonding (i.e., endogenous opioids, oxytocin, and prolactin), in the *sadness* system, which evolved over a hundred million years ago (e.g., in birds); see Panksepp (2010). The fact that *sadness* scores in the current study predicted an individuals’ distance from the group, even when accounting for other factors (musicianship, adaptivity, etc.), is perhaps indicative of the way physiological / affective states related to *sadness* affect one’s overall ability to connect with others—a situation with both psychological and neurochemical underpinnings. Indeed, oxytocin has recently been suggested to improve predictive sensorimotor abilities in dyadic tapping contexts (Gebauer et al., 2016). In general, high sadness might involve low overall arousal which may translate to less attention, motivation, or motoric responsivity in trying to align with a beat. Future studies should more directly investigate the possible links between person-level variables and tapping performance in social contexts.

A second exploratory analysis aimed to predict participants’ individual groove ratings from their SD asynchrony difference from the group. Note that we used groove ratings because groove was the scale we were most interested in and because we were on the single participant level (i.e., the factor analysis to obtain enjoyment scores reported earlier had been done on the group average level; obtaining factor scores for individuals would require re-running the factor analysis in a multi-level way, which is beyond the scope of these exploratory analyses). Groove rating was the dependent variable, individual-group SD asynchrony the independent variable, and all random effects were as specified in the previous model (adaptivity nested in participant, group, experiment). The full model is reported in Table 5.

**TABLE 5:** Individual groove ratings as a function of SD asynchrony difference from group.

Predictors	Estimates	CI	*p*
(Intercept)	0.09	0.05–0.13	< 0.001
Individual-Group SD async	−0.01	−0.01–−0.00	< 0.001
**Random effects**			
σ^2^	0.87		
τ_00 alpha:subID:group:exp_	0.07		
ICC	0.07		
N _alpha_	4		
N _subID_	284		
N _group_	71		
N _exp_	3		
Observations	6,508		
Marginal R^2^ / Conditional R^2^	0.009 / 0.080		

Individual differences in SD asynchrony from the group significantly predicted individual groove ratings, though this effect was quite small (explaining 0.9% of variance). With random effects included, the variance explained by the model increased to ∼8%. This low percentage of variance explained perhaps indicates that while individual performance in a group context is a predictor of individual subjective experience, complex subjective states, like being in the groove, are also much more than a function of individual performance when in a group context.

**FIGURE 7:**
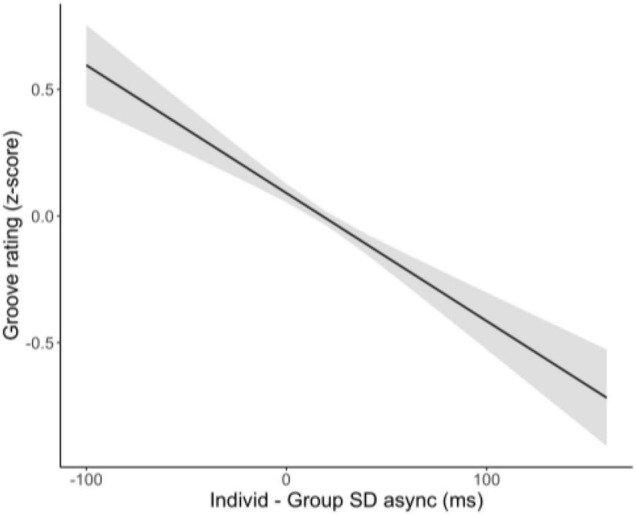
Predicted groove ratings as a function of individuals’ SD async differences from the group, adjusted for random effects of adaptivity, participant, group, and experiment (see model reported in Table 5).

**FIGURE 8:**
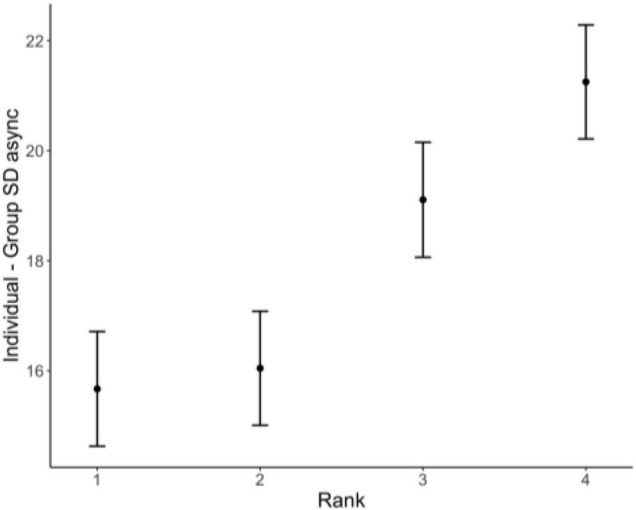
Predicted individual minus group SD asynchrony as a function of individual rank in the group, adjusted for random effects of adaptivity, participant, group, and experiment (see Table 6).

We also wish to note the asymmetry with respect to individual SD async differences: Having a negative asynchrony difference is predicted to be more groove-inducing than the opposite (positive value = less groove); see Figure 7. At first this finding may seem confusing, as one may think a difference from the group is just that, and the sign should not necessarily matter. To explore the underlying source of this important asymmetry further, we asked whether tappers with negative SD asynchronies with respect to the group tended to be the better tappers in the group. We assigned a tapper rank for each run, by sorting the tappers in the group by the absolute value of their individual minus group asynchronies. For example, if, on a given run, a group’s four tappers had the following SD asynchrony differences [10.9, −7.8, 6.5, 15.4], their ranks would be [3, 2, 1, 4]. We then compared this ranking (based on absolute value) with the signed differences. Out of the 1,627 runs across all groups for this analysis, only 424 runs (26%) had tappers exhibiting negative SD async differences. Of those 424 runs, 269 (63%) were from a tapper who ranked 1 (144/424) or 2 (125/424) for that run, suggesting there was an asymmetry in the type of tapper that tends to have a negative SD asynchrony difference to the group (N.B. there were 149 unique tappers in this pool). To further confirm the relationship between rank and signed SD asynchrony difference, we ran a linear mixed model with signed individual minus group SD async difference as the dependent variable and rank as the predictor (with all random effects as before). The overall model is significant; with rank 1 as the referent, tappers of ranks 3 and 4 have significantly higher SD asynchrony differences (see Table 6 and Figure 8).

**TABLE 6:** Tapper rank as a predictor of their SD async difference.

Predictors	Estimates	CI	*p*
(Intercept)	15.67	14.63–16.71	< 0.001
Rank [2]	0.37	−0.69–1.44	0.491
Rank [3]	3.44	2.34–4.53	< 0.001
Rank [4]	5.58	4.51–6.65	< 0.001
**Random effects**			
σ^2^	181.82		
τ_00 alpha:subID:group:exp_	159.71		
ICC	0.47		
N _alpha_	4		
N _subID_	284		
N _group_	71		
N _exp_	3		
Observations	6508		
Marginal R^2^ / Conditional R^2^	0.015 / 0.476		

Upon further reflection, an interpretation for this asymmetric finding begins to come into focus: Tappers whose individual minus group SD asynchrony is negative are more stable tappers than the group. While it was often the case that the group was more stable than any individual (all individuals with positive SD async differences), over a quarter of the time, there were some individuals who outperformed the group—such performance was associated with higher groove ratings. Future studies might more directly explore these relations, as well as the way individual vs. group performance might change as a function of group size.

## General Discussion

To probe the psychological effects of motor synchronization among individuals and groups of tappers, we developed an open-source, multi-person adaptive metronome. Across five experiments, individuals and groups tapped along with our adaptive metronome, while we manipulated both metronome adaptivity and auditory feedback. In an initial proof-of-concept experiment, we replicated the findings of Fairhurst et al. (2013) using our new device. Specifically, we showed that at optimal levels of adaptivity (25–50%), individuals achieve improved motor synchrony with the metronome, compared to non-adaptive conditions, and that subjective enjoyment can be manipulated as a function of metronome adaptivity. In a second experiment, we extended these single person tapping findings by showing synchrony with the metronome is further enhanced with the use of auditory feedback (participants hearing a sound produced by their own tap). In this experiment, asynchronies were lower in all conditions, compared to Experiment 1. Hence, depriving a person of self-auditory feedback induces a significant synchronization cost. However, auditory feedback evokes mixed effects in terms of subjective experience: no feedback results in higher subjective enjoyment when the metronome does not adapt (adaptivity = 0%); however, participants report much less enjoyment at higher levels of adaptivity, when they have no feedback. Conversely, with auditory feedback, subjective enjoyment can be enhanced *via* use of optimally adaptive metronome conditions.

In experiments 3–5, we tested synchronization abilities among groups of four people tapping together. We showed that the synchronization abilities of the group improve with both optimal levels of adaptivity and auditory feedback. Hearing oneself and/or the others in the group led to greater stability in tapping performance, compared to no feedback, especially at higher levels of adaptivity. The role of auditory feedback in improving tapping performance is in line with previous results (Goebl and Palmer, 2009; Konvalinka et al., 2010; Schultz and Palmer, 2019; N.B. these studies involve tapping continuation paradigms and do not use an adaptive metronome). Interestingly, though, while hearing everyone in the group is most similar to an actual music-making situation, and perhaps most ideal in terms of social engagement, tapping performance was best when participants could only hear themselves (not all others in the group). This finding makes sense when considering the likely perceptual interference caused by hearing others’ taps. This logic is in line with the results of a previous dyadic tapping study which showed poorer synchronization performance when tappers could hear each other (Konvalinka et al., 2010), as well as Versaci and Laje’s (2021) findings that auditory feedback decreases re-synchronization accuracy after period perturbation. While having information about others’ taps would be relevant for synchronizing appropriately with the metronome in the adaptive conditions, it is possible hearing all taps was distracting for overall performance and/or that masking occurred because all participants produced the same tap sound (Meyer, 2009). Future studies should explore whether assigning each participant a different instrument timbre affects these results. Overall, these findings point to the importance of considering the type of auditory feedback participants receive when designing future tapping studies.

With regard to subjective experience, we found that the factor structure we identified in the single person experiments replicated in the group experiments. Namely, feeling in the groove, feeling in synchrony with the metronome, feeling in synchrony with the group, and wanting to continue the task all loaded positively onto a one-factor solution, while experiencing the task as difficult loaded negatively. We termed this factor Enjoyment and found that, while Enjoyment was easy to push around in the individual tapper experiments, in group experiments, it was only with no auditory feedback and at higher levels of adaptivity that we were able to reduce subjective enjoyment. With auditory feedback, enjoyment stayed relatively flat, not moving significantly from baseline, though trending up or down with respect to optimally adaptive conditions or not. However, those enjoyment scores were averaged across the whole group.

In exploratory analyses, we related an individual’s groove ratings on any given trial to their personal SD asynchrony distance from the group on that trial. We found the lower the SD asynchrony difference, the more in the groove participants felt. We also found their SD asynchrony difference from the group was predicted by their Gold-MSI score and the *sadness* scale of the BANPS. Such exploratory findings relating individual differences to group performance and subjective experience should be more systematically explored in future studies by, for example, comparing groups of people matched/opposed in musical ability and/or some specific personality feature of interest, or, by assigning people with certain personality traits to certain musical roles. Importantly, although musical sophistication was relevant in predicting an individual’s performance, we showed individuals or groups benefited from optimal metronome adaptivity regardless of their musical abilities.

### Future Directions

We have shown that the multi-person adaptive metronome helps to bring groups of people into greater synchrony and we know from previous studies that such alignment increases social bonding and cooperative behavior. Future studies should directly investigate this connection using the adaptive metronome. For example, to further study the social utility of interacting with a multi-person adaptive metronome, one could have participants complete tasks such as the public goods game (e.g., Fischbacher and Gachter, 2010) or facial emotion recognition task (Passarelli et al., 2018) after rounds of tapping. Such experiments could additionally manipulate person-level variables to study group affiliation, for example, to study whether the adaptive metronome can enhance cohesion between people with conflicting viewpoints, identities, and/or milieus.

Others might also consider studying the effects of group size on synchronization dynamics. For example, in a previous tapping continuation task, Okano et al. (2017) showed that dyadic vs. solo tappers tend to show a greater increase in speed over time. These dynamics could now be explored in an adaptive context, with more tappers. Such medium-size group research has been suggested to be particularly fruitful for uniting large and small-scale theories of coordination dynamics, under a framework combining the Kuramoto and Haken–Kelso–Bunz equations (Kelso, 2021), or to study coordination at local vs. global timescales (Okano et al., 2019). However, we wish to note, at present, the group size with our current system is limited to four tappers because the Arduino Uno has only four input/output pins. While this hardware allows the study of group sizes from 2 to 4 people, those interested in exploring dynamics of larger groups should implement the adaptive metronome code on microcontrollers with a greater number of I/O pins.

Future studies should also experiment with different real-time adaptive algorithms. For example, it would be possible to differentially favor the best or worst participants *via* a weighted average of participant asynchrony, or, to adjust the volume of the metronome based on participant performance. Additionally, others might consider using more interesting repeating patterns for the metronome, such as the *clave son* pattern, and asking participants to tap along. It is likely more interesting rhythms will lead to even greater engagement with the task and feelings of group affiliation, as previous studies have shown rhythmic music has greater effects on prosocial behavior than a purely isochronous metronome (Stupacher et al., 2017a,b) and that rhythmic complexity modulates synchronization abilities (Mathias et al., 2020). It would also be possible to have participants freeform tap and have the metronome come in as an additional player, based on the rhythmic characteristics of the participants’ tapping, though this would require significant additional programming and perhaps a micro-controller with more random-access memory than the Arduino.

Without developing new metronome algorithms, researchers might still experiment with sonic and group dynamics, using the existing system as is. For instance, one could use different timbral or registral qualities of the metronome and tapping sounds to influence participant dynamics [see Keller and Repp (2008) for single person example]. Likewise, the attack, duration, and frequency of a sound are known to affect the perception of the center of the beat and tapping synchronization with the beat (see e.g., Hove et al., 2007; Danielsen et al., 2019); such dynamics could now be explored in a multi-person context, with sounds varying in these different features assigned to different participants and/or the metronome. Similarly, one could assign certain tappers certain roles and investigate leader-follower dynamics in dyads, triads, and quartets. As visual information has been shown to influence synchronization dynamics and groove ratings (see e.g., Tognoli et al., 2007; Dotov et al., 2021), participants’ visual information with respect to each other could also be manipulated. Single finger or bimanual tapping could be used, different metronome tempi could be investigated, and so on. Further, the effects of the multi-person adaptive metronome could be explored in groups of individuals sharing (or not) similar preferred endogenous tempi and/or musical expertise, as previous work points to the importance of both of these factors in influencing synchronization abilities (Zamm et al., 2016; Schultz and Palmer, 2019; Scheurich et al., 2020). Additionally, recent work reveals systematic differences in tapping synchronization abilities in neurodevelopmental (Vishne et al., 2021) and neurodegenerative (Janzen et al., 2019; Curzel et al., 2021) disorders; the adaptive metronome may, therefore, be a useful therapeutic device in clinical contexts.

In summary, this low-latency, low-cost, adaptive metronome system holds promise in bringing groups of people into greater motoric and psychological alignment in a variety of contexts. For example, it could be used in an assistive context, so those with difficulty perceiving and tapping to a beat, or little musical training, can easily come together to have a musical experience and feel connected, or a pedagogical context, such that those just starting out in music might experience less frustration when learning to accurately keep time and might more quickly come to experience the feeling of being in synchrony with a metronome or in the groove with others. Most importantly, the multi-person person adaptive metronome, which we internally refer to as GEM: the Groove Enhancement Machine, allows for studying, in a controlled way, the variables which may impact motor synchronization, subjective experience, and social bonding in a group context. In making the code and wiring diagram for GEM publicly available (see GitHub repository), we hope others will join us in building out the capabilities of the metronome system, and in carrying out future experiments probing the psychological and neural underpinnings of inter-personal synchronization.

## Data Availability Statement

The datasets presented in this study can be found in online repositories. All code required to program the Arduinos and create the GEM system is available at: https://github.com/janatalab/GEM/releases/tag/v1.0.0. All code required to run the experiments reported in this paper and to recreate the statistical analyses is available at: https://github.com/janatalab/GEM-Experiments-POC.

## Ethics Statement

The studies involving human participants were reviewed and approved by University of California, Davis, Institutional Review Board. The patients/participants provided their written informed consent to participate in this study.

## Author Contributions

LF: methodology, software, validation, data curation, investigation, formal analysis, visualization, and writing—original draft. PA: methodology, software, validation, data curation, formal analysis, and writing—review and editing. PJ: conceptualization, methodology, software, validation, data curation, formal analysis, visualization, writing—review and editing, supervision, resources, project administration, and funding acquisition. All authors contributed to the article and approved the submitted version.

## Conflict of Interest

The authors declare that the research was conducted in the absence of any commercial or financial relationships that could be construed as a potential conflict of interest.

## Publisher’s Note

All claims expressed in this article are solely those of the authors and do not necessarily represent those of their affiliated organizations, or those of the publisher, the editors and the reviewers. Any product that may be evaluated in this article, or claim that may be made by its manufacturer, is not guaranteed or endorsed by the publisher.

## Footnotes

1
Arduino.cc

2
Adafruit.com
