# Supplementary material for: The Groove Enhancement Machine (GEM): A Multi-Person Adaptive Metronome to Manipulate Sensorimotor Synchronization and Subjective Enjoyment
Source: Front Hum Neurosci. 2022 Jun 15;16:916551. doi: 10.3389/fnhum.2022.916551 (PMC9240653; doi:10.3389/fnhum.2022.916551)
Supplement: Supplementary file 1 [file Table_1.DOCX]

**Appendix 1:** Correlations (Pearson) between all subjective ratings in each experiment

Exp. 1

groove sync like diff infl

groove - *** *** *** **

sync 0.786 - *** *** *

like 0.664 0.566 - *** *

diff -0.769 -0.872 -0.524 -

infl 0.354 0.24 0.289 -0.186 -

Exp. 2

groove sync like diff infl

groove - *** *** ***

sync 0.5 - *** *** ***

like 0.365 0.528 - *** ***

diff -0.416 -0.614 -0.483 - ***

infl 0.121 0.299 0.438 -0.29 -

Exp. 3

groove sync_self like diff infl sync_others

groove - *** *** *** *** ***

sync_self 0.952 - *** *** *** ***

like 0.808 0.769 - *** *** ***

diff -0.914 -0.933 -0.752 - *** ***

infl 0.748 0.738 0.627 -0.695 - ***

sync_others 0.908 0.947 0.726 -0.9 0.736 -

Exp. 4

groove sync_self like diff infl sync_others

groove - *** *** ** * ***

sync_self 0.684 - ** *** ***

like 0.355 0.333 - ** *

diff -0.298 -0.485 -0.082 - **

infl 0.223 0.126 0.327 0.185 -

sync_others 0.421 0.497 0.239 -0.303 0.165 -

Exp. 5

groove sync_self like diff infl sync_others

groove - *** *** ** *** *

sync_self 0.637 - *** *** *** **

like 0.51 0.483 - ** *** ***

diff -0.381 -0.594 -0.377 - **

infl 0.44 0.475 0.424 -0.189 -

sync_others 0.288 0.349 0.416 -0.349 0.197 -

**Appendix 2:** Factor loadings for all experiments

Exp. 1

Item Factor communalities

groove 0.663033 0.439612

sync 0.835897 0.698724

like 0.460099 0.211691

diff -0.771964 0.595929

Exp. 2

Item Factor communalities

groove 0.690696 0.477061

sync 0.851973 0.725859

like 0.397344 0.157882

diff -0.688876 0.474550

Exp. 3

Item Factor communalities

groove 0.89306 0.797556

sync_self 0.936666 0.877344

like 0.517401 0.267704

diff -0.79217 0.627533

sync_others 0.823069 0.677443

Exp. 4

Item Factor communalities

groove 0.722108 0.521440

sync_self 0.878406 0.771597

like 0.391389 0.153185

diff -0.583817 0.340842

sync_others 0.634358 0.402410

Exp. 5

Item Factor communalities

groove 0.677655 0.459217

sync_self 0.885506 0.784121

like 0.49596 0.245976

diff -0.615894 0.379326

sync_others 0.602022 0.362431
